# A Review on Properties of Electrodeposited Nickel Composite Coatings: Ni-Al_2_O_3_, Ni-SiC, Ni-ZrO_2_, Ni-TiO_2_ and Ni-WC

**DOI:** 10.3390/ma17235715

**Published:** 2024-11-22

**Authors:** Daniel M. Zellele, Gulmira Sh. Yar-Mukhamedova, Malgorzata Rutkowska-Gorczyca

**Affiliations:** 1 Faculty of Physics and Technology, Al-Farabi Kazakh National University, Almaty 050040, Kazakhstan; gulmira-alma-ata@mail.ru; 2 Faculty of Mechanical Engineering, Wrocław University of Science and Technology, 50-370 Wrocław, Poland

**Keywords:** electrodeposition, nickel composite coatings, corrosion resistance, hardness, wear resistance

## Abstract

Nickel electrodeposition is a widely utilized method for creating thin films on various substrates with various desirable attributes. Recently, there has been a growing interest in developing nickel composite coatings that incorporate additional elements or particles into the nickel matrix to enhance their properties. These composite coatings offer superior corrosion resistance, hardness, tribological, and other functional benefits compared with pure nickel coatings. Some of the recent advancements in electrodeposited nickel composite coatings include improved wear resistance, enhanced mechanical properties, and better corrosion resistance. Researchers have discovered that reinforcing the nickel matrix with Al_2_O_3_, SiC, ZrO_2_, WC, and TiO_2_ particles to obtain nickel composite coatings can significantly enhance all these important functional properties of various substrates. The uniform distribution of these particles within the nickel matrix acts as a barrier to wear and tear. Studies have also shown that nickel composite coatings with those particles exhibit superior mechanical properties, including increased hardness. These particles help to refine the grain size of the nickel matrix and deter movements that may cause defects, leading to greater mechanical strength. Moreover, nickel composite coatings offer improved protection against corrosion compared with pure nickel coatings. This review provides a detailed discussion of nickel composite coatings with regard to their comparative advantages compared with pure nickel coatings on different substrates.

## 1. Introduction

Electrodeposited composite coatings offer a superior alternative to single-metal coatings, as they combine the beneficial properties of multiple materials. By incorporating dispersed phases, like ceramic nanoparticles, these coatings enhance mechanical strength, corrosion resistance, and tailored functionalities [1,2]. Ongoing advancements in material combinations and deposition techniques position composite coatings to significantly influence a broad spectrum of industrial applications. Electrodeposited nickel composite coatings are widely used in numerous industrial applications due to the fact that they possess a unique combination of properties. These coatings offer high corrosion resistance, better resistance to wear, and enhanced mechanical properties when compared with pure nickel coatings. Understanding the characteristics and properties of these composite coatings is crucial for optimizing their performance in specific applications. In this review, the aim is to explore the key characteristics of electrodeposited nickel composite coatings, including their microstructure, hardness, and adhesion properties. By gaining a deeper insight into these characteristics, we can enhance our knowledge of how these coatings function and contribute to their improved design and performance. This review offers comprehensive and valuable insights into the development and application of electrodeposited nickel composite coatings and their various properties.

The electrodeposition process of nickel composite coatings represents a significant advancement in surface modification techniques, offering enhanced properties crucial for various applications. Pulse electrodeposition and pulse reverse electrodeposition have emerged as transformative methods, enabling improved surface characteristics such as reduced porosity, minimal inclusions, and higher deposition rates compared with traditional direct current methods. These processes provide flexibility in adjusting key parameters like pulse current density, on time, and off time to tailor the composition and microstructure of the coatings. Incorporating nanoparticles, such as Al_2_O_3_, SiC, and ZrO_2_, into the nickel matrix through electrodeposition yields composite coatings with superior microhardness, wear resistance, and unique grain textures. A detailed investigation into the effects of process parameters on the mechanical properties and microstructure of these coatings reveals promising results, with Ni-Al_2_O_3_ composites exhibiting exceptional hardness and wear resistance. Furthermore, tribo-corrosion studies on NiP and NiP-SiC coatings highlight the impact of SiC particle dispersion on wear volume loss and current density during testing, emphasizing the intricate interplay between composition and performance in electrodeposited nickel composite coatings.

## 2. Electrodeposition

Electrodeposition involves the use of an applied current (potential) to deposit a film of metal or alloy onto the surface of a conductive substrate by reducing metal ions. When particulates are co-deposited with the metal or alloy, a composite is formed. This process is used to apply a thin layer of metal to a workpiece, enhancing properties such as corrosion resistance, wear characteristics, and aesthetics. By coating inexpensive base materials with layers of metals that have superior properties, electrodeposition extends their applications and makes them more cost-effective. This versatile technology offers various methods to create thin films and coatings on target substrates [3,4]. Below is a breakdown of the different electrodeposition methods.

### 2.1. Direct Current Electrodeposition

This is the simplest, most basic, and widely used method of electrodeposition. A constant direct current is applied, causing positively charged ions (cations) in the electrolyte solution to migrate toward the negatively charged electrode (cathode), where the coating is desired. At the cathode, the cations are reduced to their elemental form, depositing as a thin film on the substrate. This technique is further divided based on the electrodes’ orientation in the electrolyte bath during the process of electrodeposition, namely, conventional electro co-deposition (CECD) and sediment co-deposition (SCD). In the CECD method, the electrodes are vertically placed inside the electrolyte bath, while in SCD, horizontal placement of electrodes is used. These orientations significantly influence the adsorption of nanoparticles into the alloy matrix during electrodeposition.

In a study investigating the effect of particle size and co-deposition technique on the hardness and corrosion properties of Ni-Co/SiC composite coatings, it was found that these factors greatly impact the final properties of the coatings [5]. Here, it was found that gravitational pull and the electrophoresis force, the two main forces involved during the SCD electrodeposition method, produced higher corrosion resistance compared with conventional deposition (CECD), which exclusively relies on gravitational pull. Direct current electrodeposition has comparative advantages over other coating techniques due to its simplicity in terms of setup and operation, as well as the availability of a well-established, technical, and reliable process. Another study [6] demonstrated that DC setups are easier to design, operate, and maintain compared with those requiring precise control of pulse parameters in PC and PRC methods. The results indicate that PC electrodeposited films exhibit a porous morphology with smaller crystallite sizes and higher donor density compared with DC electrodeposited films, which feature equiaxed particles in their morphology. This simplicity of DC setups translates to lower initial investment and operational costs. Additionally, all direct current-deposited Ni and Ni nanocomposite coatings displayed significantly stronger (more uniform) crystallographic textures when compared with those deposited using pulse current (PC) and pulse reverse current (PRC) techniques [7].

**Figure 1:**
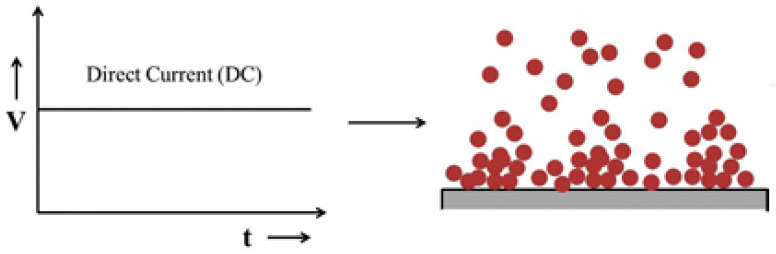
Schematic representation of DC electrodeposition and expected deposit growth, adopted from [8].

Direct current (DC) electrodeposition is a conventional method where the application of constant current (potential) is continuously used during the deposition process to coat the desired substrates. Figure 1 below illustrates the schematic diagram of the application of direct current and the distinctive growth process associated with this process. This method primarily involves two variables, namely, the applied potential (current) and the time of deposition, while precursor concentration and pH of the electrolyte in the bath are also important factors. By adjusting these parameters, the morphology, composition, and thickness of the deposited coat can be modified. In the direct current deposition, the continual application of constant potential (current) results in the deposition of the desired film without any relaxation, encouraging the growth of the already existing nuclei instead of generating new nucleation sites, which leads to the formation of a rough and porous surface deposit, as shown in Figure 1 below.

### 2.2. Pulse Electrodeposition

**Figure 2:**
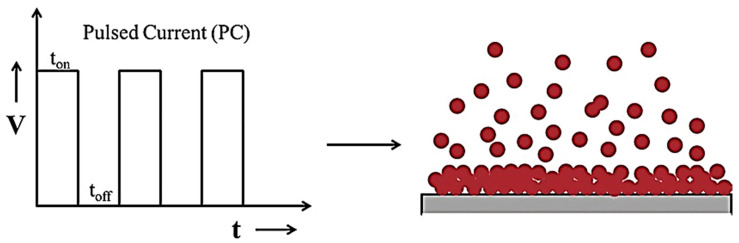
Schematic representation of PED method and expected deposit growth, adopted from [8].

This method represents an advancement in the process of electrodeposition of metals, metal alloys, and metal matrix composites (MMCs). Unlike the traditional DC electrodeposition method, PED provides enhanced flexibility in adjusting key deposition process parameters such as current density at peak (Ip), on-time pulse current (t_on_), and off-time pulse current (t_off_). This flexibility enables the creation of tailored compositions and microstructures in the deposited coatings. Achieving the desired average current density (I_a_) involves the utilization of various combinations of peak current densities, on-times of pulse currents, and off-times of pulse currents [9]. Additionally, the current in the PED method can be efficiently changed between two different values, creating a sequence of pulses with equivalent amplitude, time duration, and polarity, separated by zero current [10]. Figure 2 below illustrates a representation of the waveform of pulse current where the current oscillates between a positive current value (Ip) for the duration of t_on_ and a current value of zero (0) for the duration of t_off_.

The concepts of pulse and pulse reverse techniques are presented below:

- During the t_off_ period in PED, the electric double layer found around the cathode undergoes a discharge, allowing the ions to pass through it and reach the cathode surface. In contrast, during direct current electrodeposition, this same double electric layer hinders ions from reaching the desired surface on the cathode.
- During the process of electrodeposition, regions with high-current density inside the electrolyte bath experience greater ion depletion compared with those of low-current-density areas. Throughout the t_off_ period, the migration of ions to these low-current areas (depleted areas) takes place, ensuring a better uniform ion distribution for deposition when the t_on_ pulse happens.

**Figure 3:**
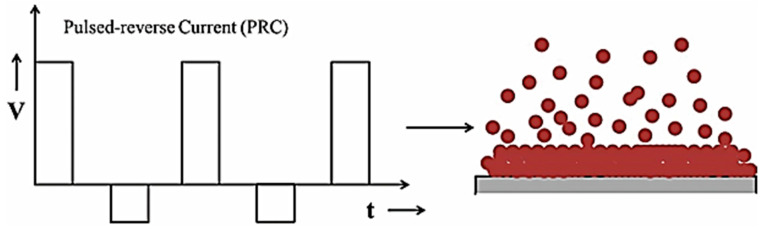
Schematic of PRC electrodeposition and expected deposit growth, adopted from [8].

The pulse electrodeposition method enables the synthesis of coatings with controlled thickness, composition, and microstructure by adjusting pulse parameters. PC (pulse current) and PRC (pulse reverse current) electrodeposition, shown in Figure 3, techniques offer several advantages and disadvantages compared with the DC electrodeposition technique [4,11]. The advantages are as follows:

- Replenishing metal ions in the diffusion layer during the off time significantly increases the limiting current density.
- Greater flexibility in pulse parameters minimizes process constraints.
- Leads to the formation of fine-grained deposits, which display a tighter structure with reduced porosity and experience less stress.
- Enhances deposit adhesion and ensures a consistent uniform thickness.
- Increases deposition rate while enhancing physical and mechanical properties.
- On the other hand, the pulse electrodeposition method has downsides:
- Expensive pulse generators compared with DC units.
- It requires careful advance planning and a sequence of working procedures to achieve optimal outcomes.

### 2.3. Jet Electrodeposition

**Figure 4:**
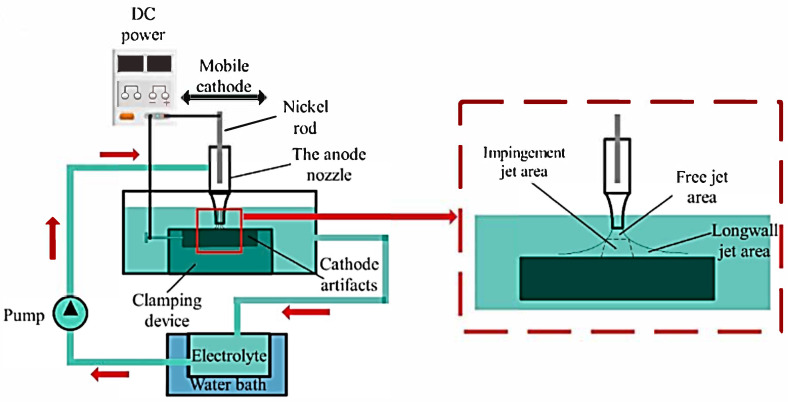
Schematic diagram for typical jet electrodeposition process, adopted from [12].

Jet electrodeposition is a technique used for precise metal deposition, particularly on small or intricate surfaces. It operates by directing a high-velocity jet of electrolyte solution containing metal ions onto a target surface (the cathode). The electrolyte jet is typically delivered through a fine nozzle, allowing for localized deposition. When a voltage is applied between the nozzle (which acts as the anode) and the cathode, metal ions in the solution are reduced and deposited onto the cathode surface. This method allows for high deposition rates and enhanced control over the thickness and uniformity of the metal layer. The high flow rate of the electrolyte also helps remove unwanted byproducts, maintaining optimal conditions for electrodeposition and enabling the fabrication of fine structures or coatings. Figure 4 below illustrates the setup configuration of jet electrodeposition.

As the plating electrolyte solution flows, electric potential is conducted through the solution to the substrate surface, facilitating deposition on the cathode surface where the jet flows over. The jet electrodeposition method is a rapid coating technique that offers numerous advantages over other coating methods, such as high deposition rates and effective refinement of grain size [13,14].

### 2.4. Ni-Al_2_O_3_ Composite Coatings

Electrodeposited Ni composite coatings incorporating ceramic particles have been extensively studied for their enhanced mechanical strength, wear resistance, and corrosion protection compared with conventional nickel coatings [15,16,17,18]. Ni-Al_2_O_3_ composite coatings, consisting of nickel (Ni) and alumina (Al_2_O_3_), have emerged as a powerful technology for enhancing the properties of underlying substrate materials. These coatings offer a unique blend of properties derived from their individual constituents. Nickel provides excellent ductility, strength, and electrical conductivity, while alumina contributes superior wear resistance, high-temperature stability, and outstanding corrosion resistance [19]. Consequently, this synergy makes Ni-Al_2_O_3_ coatings ideal for various applications demanding protection against harsh environments. It is observed in [20] what effect does current density bring on electrodeposited Ni-Al_2_O_3_ composite coatings. Wear resistance, as well as the microhardness of these composite coatings, showed a significant improvement at a current density of 0.01A/dm^2^. This significant improvement in hardness was primarily due to the combined effects of strengthening of dispersion, as well as refined grain sizes. Besides, the abrasive strength of this composite coating was found to be 57 MPa. By varying alumina content in the electrolyte bath, improved wear and corrosion resistance was achieved as well. Here, upon increasing Al_2_O_3_ particles in the composite coating, the wear rate of the coatings happens to decrease from 5.96 × 10^−5^ to 3.16 × 10^−5^ mm^3^ /Nm. This indicates an approximate improvement of twice as much in the wear resistance. At the same time, the resistance to corrosion was also increased with increasing particle concentration, resulting in improved corrosion from 392 value to 063 µm/year value. It was also found in [21] that, with the pulse electrodeposition method, the roughness properties of Ni-Al_2_O_3_ composite coatings were improved with varying duty cycles from 20 to 100%. The study at hand investigates the impact of duty cycle variations on the surface roughness properties of substrates in pulse coating processes. The duty cycle, defined as the ratio of the pulse duration to the total cycle time, plays a critical role in determining the final characteristics of the coated surface. The results of the study reveal a significant inverse relationship between the duty cycle and the enhanced surface roughness properties of the substrate. The surface roughness is a critical parameter that affects the functionality and aesthetics of coated materials. In the study, surface roughness was quantified by measuring the average roughness, with values ranging from 0.779 µm to 0.245 µm. The results demonstrate that decreasing the duty cycle leads to a marked improvement in surface roughness. The enhancement in surface roughness, as the duty cycle decreases, can be attributed to changes in the grain size of the coating material. Pulse coating processes involve the intermittent application of material onto a substrate, which is followed by periods of relaxation or cooling. The duty cycle determines the proportion of time the substrate is exposed to the coating material versus the time it is not. A lower duty cycle means that the substrate is exposed to the coating material for a shorter period within each cycle. This reduced exposure time results in finer grains, as there is less time for the material to coalesce into larger grains. On the contrary, a higher duty cycle allows for longer exposure, which leads to the formation of larger grains. The grain size is inversely proportional to the duty cycle; thus, a decrease in the duty cycle results in a finer microstructure with smaller grains. Likewise, smaller grain sizes contribute to a smoother surface due to the fact that the material deposits more uniformly when the grains are finer. This fine-grain structure leads to a reduction in the peaks and valleys on the substrate surface, thereby improving surface smoothness. In contrast, a higher duty cycle with larger grains tends to produce a rougher surface due to the more pronounced irregularities created by the larger grains. Moreover, during pulse coating, each cycle of coating application and cooling affects the morphology of the deposited material. Lower duty cycles reduce heat accumulation and allow for rapid cooling, which can further refine the grain structure and contribute to smoother surfaces. On the other hand, higher duty cycles increase heat and allow for more substantial grain growth before cooling occurs, resulting in increased surface roughness. Therefore, optimizing the duty cycle in pulse coating processes is crucial for achieving desired surface roughness properties. In so doing, we can influence the grain size and, consequently, the smoothness of the coated substrate. A lower duty cycle is shown to be beneficial for enhancing surface roughness, evidenced by the reduction in roughness values from 0.779 µm to 0.245 µm. This finding underscores the importance of precise parameter control in pulse coating techniques to achieve high-quality surface finishes.

Besides the concentration of particles, studies show the hardness, wear resistance microstructure, and surface morphology of composite coatings are highly influenced by current density as well [22]. With increasing current density, the microhardness values of deposits were found to increase. This may be due to the higher amounts of incorporated alumina particles in the matrix. Upon increasing the current density from 3 A/dm^2^ to 5 A/dm^2^, the value of polarization resistance improved, and the corrosion current density decreased. Moreover, the corrosion potential of the samples appeared to shift more toward the positive side, which indicated an improvement in corrosion resistance. It was observed that the higher the current density the coating was deposited, the more homogeneous distribution of the ceramic phase. The smaller values of residual stresses revealed that the deposition is characterized by smaller corrosion current density and higher polarization resistance.

In Ni-Al_2_O_3_ composite coatings, the alumina (Al_2_O_3_) content significantly influences the overall corrosion resistance of the substrate, primarily due to the distinct properties of alumina as a ceramic material. Alumina is well-known for its high hardness, chemical inertness, and excellent stability in corrosive environments, which contribute to its ability to enhance the protective characteristics of coatings when incorporated into a nickel (Ni) matrix. As the content of alumina increases in the composite, key effects such as barrier enhancement, improved coating hardness, corrosion potential shift, thermal stability, and microstructural refinements occur. The content of alumina plays this pivotal role in the corrosion resistance of the substrates in such a way that with increasing content of alumina, the coating’s corrosion rate declines [23]. It was observed that increasing the applied current beyond 50 mA reduced its impact on the corrosion rate. Specifically, the degradation potential became higher for the coating developed at a current of 60 mA compared with other composite coatings. Additionally, increasing the bath temperature up to 40 °C resulted in a reduction in the corrosion current density, which indicates an improvement in corrosion resistance. However, when the temperature exceeded 40 °C, the corrosion current density increased, leading to a decrease in the corrosion resistance of the composite coatings.

The microstructure and wear resistance of nickel–alumina composite coatings are significantly affected by the applied pulse frequency during the coating process. At lower pulse frequencies, these coatings tend to exhibit enhanced hardness and superior wear resistance in comparison with those produced at higher frequencies. This improvement at lower frequencies is primarily attributed to the more refined and homogeneous microstructure that develops under these conditions, which contributes to the material’s mechanical strength.

The wear resistance of these composite coatings is largely governed by the microstructural characteristics rather than solely by the presence of alumina reinforcement particles. While the alumina particles are intended to enhance the wear resistance, the overall microstructural integrity, such as grain size, phase distribution, and bonding strength between the nickel matrix and the alumina particles, plays a more crucial role. Adhesive wear, which is a common wear mechanism in these coatings, is particularly influenced by the microstructure, as it dictates how well the coating can resist material removal during sliding or abrasive contact. Therefore, optimizing the microstructure at lower pulse frequencies can lead to a more effective enhancement of wear resistance, overshadowing the direct impact of alumina particle reinforcement alone [24].

### 2.5. Ni-SiC Composite Coatings

Ni-SiC composite coatings have emerged as a promising material for surface engineering applications due to their ability to combine the desirable properties of nickel (Ni) with the exceptional hardness and wear resistance of silicon carbide (SiC) [25,26].

Electrodeposition is a widely used technique for creating Ni-SiC coatings. Recent studies have explored optimizing this process to achieve better control over SiC particle distribution and content within the nickel matrix. For instance, research by [27] investigated the use of binary nonionic surfactants to improve the incorporation of SiC nanoparticles during electrodeposition. Their findings demonstrated that surfactants improved the dispersion of SiC nanoparticles, leading to more uniform and higher hardness coatings compared with pure nickel coatings.

The primary motivation for incorporating SiC nanoparticles into nickel is to improve its mechanical properties. Studies have shown that Ni-SiC composites, which consist of nickel (Ni) as the matrix and silicon carbide (SiC) particles as a reinforcing phase, have been extensively studied due to their superior mechanical and functional properties compared with pure nickel coatings. The combination of nickel’s inherent properties with the hardness and stability of SiC particles creates a composite that outperforms pure nickel coatings in several key areas, including hardness, wear, and corrosion resistance. These enhancements make Ni-SiC composites highly suitable for applications that demand durability and protection, such as in the automotive, aerospace, and manufacturing industries, where surfaces are exposed to high friction, corrosive environments, or mechanical stress. For instance, a study carried out to investigate the effect of SiC nanoparticle concentration on the properties of Ni-SiC coatings fabricated via high-frequency inductive cladding [28] revealed that increasing the SiC content resulted in a significant improvement in wear and corrosion resistance, making these coatings ideal for protecting carbon steel components. This method offers rapid deposition rates but requires further investigation into optimizing parameters for achieving uniform and well-distributed SiC particles.

The microstructure of Ni-SiC composites plays a crucial role in determining their performance. The size, distribution, and interfacial bonding between Ni and SiC particles significantly impact the mechanical properties. Studies by [28] employed electrodeposition to fabricate Ni-SiC coatings with varying SiC particle concentrations. They observed that increasing the SiC content led to grain refinement and enhanced corrosion resistance in the composite coating. The incorporation of SiC nanoparticles into the nickel matrix generally improves the microhardness and wear resistance of the coating. Research by [27] showed a significant increase in microhardness compared with pure nickel coatings. This improvement can be attributed to the presence of hard SiC particles that act as load-bearing reinforcements within the nickel matrix.

Recent research efforts in Ni-SiC composites focus on improving the control over deposition processes and understanding the effect of SiC particle characteristics (size, morphology) on the final properties of the coating. Additionally, researchers are exploring novel fabrication techniques like laser cladding to achieve superior properties and address limitations associated with conventional methods.

### 2.6. Ni-ZrO_2_ Composite Coatings

Nickel (Ni) and zirconium dioxide (zirconia, ZrO_2_) have good compatibility due to their similar thermal expansion coefficient and elastic modulus. Several techniques are employed to create Ni-ZrO_2_ composite coatings. Electrodeposition is a widely used method, allowing for the incorporation of ZrO_2_ particles within a nickel matrix [29,30,31]. Studies have explored optimizing the ZrO_2_ content in the plating bath to achieve a balance between particle incorporation and distribution within the coating [29]. Sol-enhanced electroplating has also been investigated, demonstrating improved dispersion of ZrO_2_ nanoparticles compared with traditional powder addition methods [32].

Recent research highlights the importance of achieving a uniform distribution of ZrO_2_ particles within the nickel matrix [29,32]. This distribution is influenced by the ZrO_2_ concentration in the fabrication process and can significantly impact properties like hardness and wear resistance. The incorporation of ZrO_2_ into nickel coatings leads to substantial improvements in various properties. A key area of focus is enhanced wear resistance, achieved through the dispersion of hard ZrO_2_ particles within the softer nickel matrix [32,33]. Additionally, Ni-ZrO_2_ composites demonstrate improved corrosion resistance compared with pure nickel coatings [29]. The study explores the impact of ZrO_2_ concentration in the electrolytic bath on the particle distribution and corrosion resistance of the coatings. Their findings suggest an optimal ZrO_2_ concentration for achieving the best combination of these properties. This enhancement is attributed to the barrier effect of ZrO_2_ particles, hindering the diffusion of corrosive species towards the substrate. It was also found out that the microhardness and tribo-corrosion properties of composite coatings were significantly improved due to the reinforcement of uniformly dispersed ZrO_2_ particles in the nickel matrix, followed by the combined effect of dispersion strengthening and structural modification [34].

Current research efforts in Ni-ZrO_2_ composites are directed towards optimizing fabrication techniques for superior control over microstructure and achieving the desired balance between different properties. Sol-enhanced electroplating is a promising approach for achieving better dispersion of ZrO_2_ nanoparticles [32]. A sol-enhanced electroplating method has been investigated, demonstrating superior improvements in mechanical properties compared with traditional ZrO_2_ powder incorporation. Furthermore, studies are exploring the influence of ZrO_2_ particle size and morphology on the overall performance of the composite coatings.

Ni-ZrO_2_ composite coatings hold immense potential for various industrial applications due to their combined advantages of good mechanical properties, corrosion resistance, and compatibility with different substrates. Further research focused on optimizing fabrication techniques, exploring alternative ZrO_2_ dopants, and investigating the tribological behavior of these coatings will pave the way for their wider adoption in fields like automotive, aerospace, and machine tools.

The microstructure of Ni-ZrO_2_ composite coatings plays a crucial role in determining their performance. Studies [32,33] utilize Scanning Electron Microscopy (SEM) and X-ray Diffraction (XRD) to analyze the distribution and crystallographic phases within the coatings. These studies emphasize the importance of achieving a uniform dispersion of ZrO_2_ nanoparticles within the Ni matrix for optimal properties.

The primary advantage of Ni-ZrO_2_ composite coatings lies in their enhanced functionalities compared with individual materials. A study by [35] demonstrates a significant improvement in wear resistance with increasing ZrO_2_ content in the coatings. Similarly, other research highlights the enhanced corrosion resistance achieved through Ni-ZrO_2_ composite coatings [29,36,37].

### 2.7. Ni-TiO_2_ Composite Coatings

Ni-TiO_2_ composite coatings have gained significant attention in recent years due to their potential applications in various industrial sectors. These coatings consist of a mixture of nickel (Ni) and titanium dioxide (TiO_2_) nanoparticles, which are deposited onto a substrate surface using techniques such as electrodeposition or chemical vapor deposition. The unique combination of properties offered by both Ni and TiO_2_, such as corrosion resistance, mechanical strength, and photocatalytic activity, makes these composite coatings highly versatile and suitable for a wide range of applications.

One of the key advantages of Ni-TiO_2_ composite coatings is their enhanced corrosion resistance compared with traditional coatings. The addition of TiO_2_ nanoparticles improves the overall chemical stability of the coating, making it more resistant to harsh environments and corrosive substances [38]. This property is particularly beneficial in industries such as aerospace, automotive, and marine, where components are constantly exposed to corrosive elements. Additionally, the photocatalytic activity of TiO_2_ nanoparticles can also provide self-cleaning properties to the coating, making it easier to maintain and prolonging its lifespan.

In a comprehensive study aimed at exploring the influence of pulse electrodeposition parameters on the characteristics of nickel–titania (Ni-TiO_2_) composite coatings electrodeposited from a Watts bath, several important trends were observed. One of the key findings was that the microhardness of the composite coatings showed a distinct variation with changes in current density. Precisely, as the current density increases from 2 A/dm² to 5 A/dm², the microhardness property showed a significant enhancement. Beyond this threshold, however, further increases in current density led to a decline in the microhardness value of the coatings.

This indicates that there is an optimal current density range where the electrodeposited coatings achieve maximum hardness, beyond which the quality of the deposit begins to deteriorate. Moreover, the study identified that the composite coatings attained their highest microhardness and particle reinforcement levels when deposited at a pulse frequency of 10 Hz and a duty cycle of 10%. This combination of parameters created favorable conditions for a uniform and densely reinforced coating structure, leading to superior mechanical properties. These findings underscore the critical role that both current density and pulse electrodeposition parameters, such as frequency and duty cycle, play in determining the final properties of nickel–titania composite coatings [39].

Furthermore, the mechanical strength of Ni-TiO_2_ composite coatings makes them suitable for applications where wear and abrasion resistance are crucial. The Ni component provides excellent adhesion to the substrate surface, while the TiO_2_ nanoparticles act as reinforcements, improving the overall hardness and durability of the coating [40]. This makes them ideal for use in components subjected to high levels of mechanical stress, such as cutting tools, bearings, and medical implants. Overall, the unique combination of properties offered by Ni-TiO_2_ composite coatings makes them a promising material for various industrial applications.

### 2.8. Ni-WC Composite Coatings

Ni-WC composite coatings have gained significant attention in recent years due to their exceptional mechanical and tribological properties. These coatings are formed by incorporating tungsten carbide (WC) particles into a nickel (Ni) matrix, resulting in a material that combines the hardness of WC with the ductility of Ni. The unique combination of properties exhibited by Ni-WC composite coatings makes them ideal for a wide range of industrial applications, including cutting tools, wear-resistant surfaces, and protective coatings.

One of the key advantages of Ni-WC composite coatings is their exceptional hardness and wear resistance. Tungsten carbide is one of the hardest materials known, with a Mohs hardness of 9, while nickel provides the necessary toughness and ductility to prevent cracking and delamination. This combination of hardness and toughness makes Ni-WC composite coatings highly resistant to wear, abrasion, and erosion, making them ideal for applications in which components are subjected to severe mechanical stresses. Besides their excellent mechanical properties, Ni-WC composite coatings also show excellent corrosion resistance properties [41,42]. The nickel matrix in these coatings acts as a barrier to prevent corrosive elements from reaching the substrate material, while the tungsten carbide particles provide additional protection against chemical attack. As a result, components coated with Ni-WC composites are able to withstand harsh operating environments, such as those found in the aerospace, automotive, and oil and gas industries.

Another key advantage of Ni-WC composite coatings is their versatility and ease of application. These coatings can be deposited using a variety of techniques, including thermal spray, electroplating, and chemical vapor deposition, allowing for customization based on the specific requirements of the application [42]. Additionally, the composition and microstructure of Ni-WC coatings can be tailored to optimize properties such as hardness, wear resistance, and corrosion resistance, making them highly versatile and adaptable to a wide range of applications. Despite their many advantages, Ni-WC composite coatings have some limitations that must be considered. For example, the high hardness of tungsten carbide particles can result in increased tool wear during machining and grinding processes, which may require specialized equipment and techniques for processing. Additionally, the high cost of tungsten carbide can make Ni-WC coatings more expensive than alternative coating materials, which may limit their use in cost-sensitive applications [43].

Generally, Ni-WC composite coatings offer a unique combination of mechanical, tribological, and corrosion-resistant properties that make them ideal for a wide range of industrial applications. Their exceptional hardness, wear resistance, and corrosion resistance, coupled with their versatility and ease of application, make Ni-WC coatings a highly attractive option for components subjected to severe mechanical and environmental stresses. While they do have some limitations, the many advantages of Ni-WC composite coatings make them a valuable and promising material for future research and development in the field of surface engineering.

### 2.9. Properties of Nickel Composite Coatings

Nickel composite coatings have attracted widespread attention across numerous industries thanks to their exceptional properties and versatile applications. As researchers continue to delve into this field, numerous recent publications have shed light on the advancements and discoveries related to the properties of nickel composite coatings. Nickel composite coatings are composed of a nickel matrix with dispersed second-phase particles, including Al_2_O_3_, Si_3_N_4_, SiC, Cr_2_O_3_, WC, TiO_2_, diamond, PTFE, graphite, or even microcapsules containing liquid. Research shows these particles enhance the mechanical, tribological, and corrosion resistance properties of the composite coatings. They serve as physical barriers to dislocation movement and grain boundary sliding, resulting in a significant improvement in the mechanical properties of the composite coatings.

By incorporating a second phase, typically nonmetallic particles, into a nickel matrix, nickel composite coatings offer improved properties compared with conventional nickel coatings. Here are some key properties of nickel composite coatings.

#### 2.9.1. Hardness

Hardness is a measure of a material’s resistance to localized plastic deformation, such as indentation or scratching, when subjected to mechanical forces like pressing or abrasion [44]. Hardness is an important property in materials science and engineering due to the fact that it is directly related to a material’s performance and fit for various applications. Hardness is generally related to tensile stress yield resulting from a known amount of deformation. Nickel composite coatings reinforced with Al_2_O_3_ [45], SiC [46], and TiO_2_ [47,48] showed improved microhardness properties.

#### 2.9.2. Corrosion Resistance

Nickel composite coatings are known for their corrosion resistance properties. These coatings are used to protect various components from corrosion in a wide range of environments, including offshore and harsh chemical environments. Electrodeposited Ni-Al_2_O_3_ composite coatings consist of a nickel matrix embedded with Al_2_O_3_ (alumina) particles. This composite structure helps enhance the mechanical and chemical properties of the coating, including tribological properties [49], hardness [15] and, importantly, corrosion protection [23,49,50]. The wetting properties of these coatings play a critical role in how they interact with water and other corrosive agents due to the fact that the wetting properties of electrodeposited Ni-Al_2_O_3_ composite coatings are pivotal to their performance in anticorrosion applications. By optimizing surface energy, roughness, and alumina content, these coatings can be tailored to exhibit superior hydrophobicity, reducing water penetration and extending the material’s lifespan in corrosive environments. Thus, understanding and controlling wettability is a key factor in maximizing the effectiveness of these advanced coatings [51]. Studies show Nickel composite coatings exhibit better corrosion resistance than pure nickel coatings. It was found that increasing Al_2_O_3_ concentration resulted in a halving of corrosion rates, demonstrating that Al_2_O_3_ particle incorporation is effective in enhancing corrosion resistance [52]. By varying the concentrations of Al_2_O_3_ on steel substrates from the electroplating bath, tests conducted under working conditions demonstrated that adding Al_2_O_3_ particles (0 g/L, 20 g/L and 30 g/L) to the bath positively affects the electrochemical behavior of the steel, reducing its susceptibility to corrosion, and the optimal concentration was found to be 20 g/L [50]. The corrosion tests of the steel substrates were carried out in two different corrosive solutions of 0.5 M K_2_SO_4_ and 0.5 M NaCl, followed by examining electrochemical properties of the composite coating using potentiodynamic methods, as well as electrochemical impedance spectroscopy (EIS), while the structural and morphological analyses were carried out using X-ray Diffraction (XRD) and Scanning Electron Microscopy (SEM). At a concentration of 0 g/L Al_2_O_3_, the corrosion rate is significantly higher (7.398 mm.an^−1^) in NaCl compared to K_2_SO_4_ suggesting that the chloride (Cl^−^) ions from NaCl are more aggressive in causing corrosion compared to sulphate ions (SO_4_^2−^). The addition of 20 g/L of Al_2_O_3_ drastically reduces the corrosion current density (from 0.244 mA.cm^−2^ at 0 g/L to 0.024 mA.cm^−2^) and the corrosion rate (from 2.86 mm.an^−1^ to 0.277 mm.an^−1^) in K_2_SO_4_ by an about an order of magnitude while having a minimal effect on the values of NaCl. This suggests Al_2_O_3_ provides a more protective effect against corrosion in K_2_SO_4_ but not as much in NaCl. Further increasing the concentration of Al_2_O_3_ to 30 g/L resulted in a substantial increment of corrosion rate in both solutions, particularly in NaCl, when the corrosion rate reaches to 12.433 mm.an^−1^. This suggests that, beyond a certain conecentration of Al_2_O_3_ (20 g/L), its effectiveness as a protective agent decreases, especially in NaCl where the chloride ions dominate corrosion behavior, possibly due to changes in the electrochemical dynamics at higher concentrations. Therefore, 20 g/L of Al_2_O_3_ seems to be optimal for reducing corrosion rates [50].

Electrodeposited Ni-SiC composite coatings have shown enhanced wear and corrosion resistance as well [53]. In the study, optimal corrosion resistance values were obtained at values of 1.13 × 10^−3^ mA/cm^2^ (I_corr_) and −0.311 V (E_corr_). Another study [54] revealed that Ni-SiC composite coatings prepared on stainless steel 410 by pulse electrodeposition method produced the best corrosion resistance at a coating time of 60 min.

Studies also show that Ni-ZrO_2_ composite coatings are known for their excellent corrosion resistance, making them suitable for various industrial applications. Incorporating ZrO_2_ (zirconia) particles into a Ni (nickel) matrix enhances the coating’s properties. Research comparing Ni-ZrO_2_ composite coatings with pure Ni coatings or other composite coatings (such as Ni-Al_2_O_3_) has shown that Ni-ZrO_2_ coatings generally exhibit superior corrosion resistance due to the combined effects of the nickel matrix and the zirconia particles. In a study conducted to assess the mechanical, microstructural, and corrosion characterization of electroless Ni-P composite coatings modified with ZrO_2_ reinforcing nanoparticles, it was found that the introduction of ZrO_2_ particles enhances the corrosion resistance of coatings [55]. Moreover, Ni composite reinforced with the addition of TiO_2_ [56] and WC [57] showed enhanced corrosion resistance when compared with pure Ni coatings on various substrates. Here, the corrosion property of low-carbon steel electrodeposited with pure Ni and Ni-TiO_2_ composite coatings was investigated in a corrosive solution of 3.5 wt% NaCl at room temperature. It was found that TiO_2_ particles in the Ni matrix provided significant improvements in corrosion resistance and mechanical properties such as hardness and abrasion resistance. Furthermore, increasing WC particle concentration in the Ni matrix during the deposition process of copper substrates results in increased microhardness and roughness properties of deposits [57]. Besides these results, corrosion tests conducted in both 3.5% NaCl and 0.1 M HCl solutions offered optimum results.

#### 2.9.3. Wear Resistance

**Figure 5:**
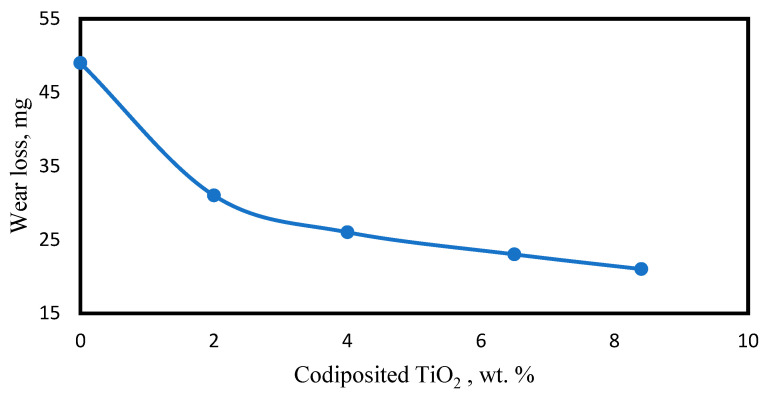
Variation in wear loss as wt.% of TiO_2_ increase, adopted from [66].

**Figure 6:**
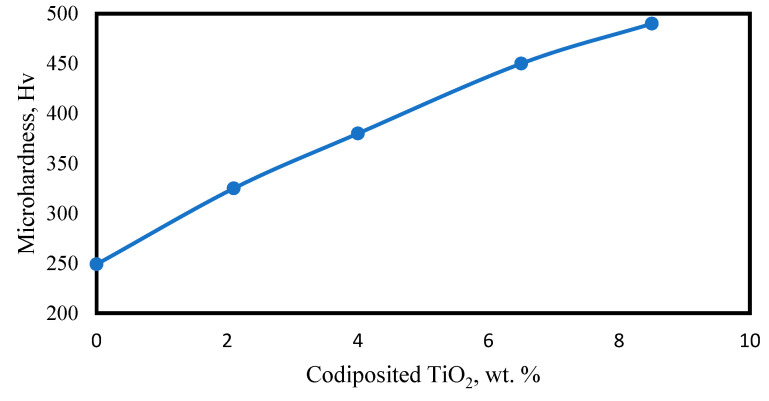
Variation in microhardness values as wt. % of TiO_2_ increases, adopted from [66].

Nickel composite coatings reinforced with Al_2_O_3_ [17,58], WC [42,59,60], TiO_2_ [40,61,62], ZrO_2_ [63,64], and SiC [25,46,65] showed enhanced resistance to wear properties when compared with pure Ni coatings on substrates. Optimal amounts of alumina particles in the electrolytic bath during Ni-Al_2_O_3_ composite coatings on steel samples made by electrodeposition have shown a more compact structure and smaller grain size in comparison with pure Ni coatings, which apparently helps to significantly improve the microhardness and wear resistance of steel substrates [58]. Here, pure Ni coating was found to possess a higher adhesion wearing rate, while the best results, only 18.5% of those of pure Ni coatings, with mild abrasive wear were obtained with the optimal (20 g/L) addition of alumina. Another study also showed that the wear rate of nickel–alumina composite coatings can be greatly reduced with optimal process parameters [17]. Electrodeposited Ni-WC composite coatings are famous for their superior hardness and wear resistance properties compared with uncoated substrates [57]. The study showed that a standard ball-on-disk wear test carried out on titanium substrate showed a significant improvement after the substrate was coated with composite Ni-WC. The wear rate of the uncoated titanium substrate was 7.034 × 10^−4^, while this figure was reduced to 2.6 × 10^−5^ mm^3^N^−1^m^−1^ after coating. This shows a notable improvement in wear resistance. Studies also demonstrated that Ni-TiO_2_ coatings exhibit excellent wear resistance and hardness properties when compared with pure nickel coatings due to the fact that the incorporation of TiO_2_ particles enhances the hardness and reduces the wear of coatings. Microhardness and wear resistance of composite coatings improve upon increasing the TiO_2_ particle content in the bath [66]. When the wear behavior of DC electrodeposited pure Ni and Ni-TiO_2_ composite coatings were evaluated using a pin-on-disk tribometer following analysis of the worn-out surface using Scanning Electron Microscopy (SEM), it was revealed that the composite coatings’ microhardness values improved as TiO_2_ content was increased from 0 to 8% wt. Similarly, friction coefficients decreased from 1 to 0.3 when the TiO_2_ content was increased from 0 to 8.3 wt. %, resulting in a decrease in wear loss from 50 mg to 8.5 mg, as shown in Figure 5 and Figure 6 below.

Ni-ZrO_2_ composite coatings are another class of nickel-based composite coatings with improved wear resistance compared with pure Ni coatings. Research conducted to study the effect of incorporating ZrO_2_ particles on a steel substrate revealed that these particles help enhance and refine the microstructure, improve the hardness, reduce the friction coefficient, and improve the temperature resistance of the coatings [64]. Furthermore, electrodeposited Ni-SiC composite coatings on copper substrate also showed improved wear resistance and hardness properties [46]. The wear resistance and hardness values of these coatings increased upon increasing Ni-SiC content. The enhancement in microhardness and wear resistance of Ni composite coatings primarily depends on the added reinforcing particles within the nickel matrix. These particles serve as a physical barrier, inhibiting the growth of nickel grain and reducing plastic deformation under various loads. This apparently promotes grain refinement and a dispersive strengthening effect, thereby improving the microhardness and wear resistance of the composite coatings [67].

### 2.10. Applications

Electrodeposited nickel composite coatings, incorporating various ceramic particles like Al_2_O_3_, SiC, ZrO_2_, TiO_2_, and WC, offer a unique combination of properties such as enhanced hardness, wear resistance, corrosion resistance, and high-temperature stability. These coatings find widespread applications in diverse industries. Below, Table 1 shows an outline of the applications of various electrodeposited nickel composite coatings.

### 2.11. Conclusions and Future Outlooks

Nickel electrodeposition is a widely used technique for producing thin films with a variety of desirable properties. In recent years, interest in nickel composite coatings has surged, driven by efforts to incorporate additional elements or particles into the nickel matrix to enhance its properties. This approach aims to improve characteristics such as durability, corrosion resistance, and mechanical strength, making these coatings more versatile and effective for a wide range of applications. These composite coatings can offer improved wear resistance, corrosion resistance, hardness, and other functional properties compared with pure nickel coatings. Future research and developments in this regard should focus more on optimizing process parameters, composition, and deposition methods to further improve the properties of these composite coatings on various substrates. Some of the recent advancements in electrodeposited nickel composite coatings include the following:

- Improved wear resistance: Researchers have found that incorporating Al_2_O_3_, SiC, ZrO_2_, WC, and TiO_2_ particles into nickel coatings can significantly improve wear resistance. The uniform distribution of the particles within the nickel matrix acts as a barrier to wear and tear.
- Enhanced mechanical properties: Studies have shown that electrodeposited nickel composite coatings with Al_2_O_3_, SiC, ZrO_2_, WC, and TiO_2_ particles exhibit superior mechanical properties, including increased hardness. The presence of these particles refines the grain size of the nickel matrix and hinders the movement of defects, leading to enhanced mechanical strength.
- Better corrosion resistance: Nickel composite coatings can offer improved protection against corrosion compared with pure nickel coatings.

## Figures and Tables

**Table 1:** Applications and properties of various Ni composite coatings.

Nickel Composite Coating	Applications	Key Properties for Application	References
Ni-Al_2_O_3_	Aerospace components Automotive engine parts Cutting tools Wear-resistant industrial components High-temperature applications	Improved wear resistance Enhanced hardness Higher thermal stability Resistance to oxidation at high temperatures	[17,68]
Ni-SiC	Precision machinery Automotive components (pistons, cylinders) Engine parts Coatings for molds and dies Textile machinery Electrical contacts	Superior resistance to wear Improved hardness and lower friction Enhanced corrosion resistance	[69,70]
Ni-ZrO_2_	Aerospace components Corrosion resistant coatings High-temperature applications (turbines, fuel cells) Structural ceramics	Higher temperature stability Better corrosion resistance Improved toughness and wear resistance	[36,71]
Ni-TiO_2_	Anticorrosion coatings Agricultural machineries Medical devices Self-cleaning and photocatalytic coatings Electronics	Excellent resistance to corrosion Self-cleaning and photocatalytic properties Enhanced mechanical properties	[38,56,72,73,74,75]
Ni-WC	Cutting and drilling tools Wear resistant coatings for mining equipment Abrasive environments (oil and gas, mining) Machine components subjected to high friction	Extremely hard and wear resistant Highly durable in erosive and abrasive conditions Better resistance to high temperatures	[76,77,78,79,80,81]
